# Short-Term Blockade of Pro-Inflammatory Alarmin S100A9 Favorably Modulates Left Ventricle Proteome and Related Signaling Pathways Involved in Post-Myocardial Infarction Recovery

**DOI:** 10.3390/ijms23095289

**Published:** 2022-05-09

**Authors:** Raluca Maria Boteanu, Viorel-Iulian Suica, Elena Uyy, Luminita Ivan, Aurel Cerveanu-Hogas, Razvan Gheorghita Mares, Maya Simionescu, Alexandru Schiopu, Felicia Antohe

**Affiliations:** 1 Department of Proteomics, Institute of Cellular Biology and Pathology “N. Simionescu” of the Romanian Academy, 050568 Bucharest, Romania; raluca.haraba@icbp.ro (R.M.B.); viorel.suica@icbp.ro (V.-I.S.); elena.uyy@icbp.ro (E.U.); luminita.radulescu@icbp.ro (L.I.); aurel.cerveanu-hogas@icbp.ro (A.C.-H.); maya.simionescu@icbp.ro (M.S.); 2 Department of Pathophysiology, University of Medicine, Pharmacy, Sciences and Technology of Targu Mures, 540142 Targu Mures, Romania; razvan.mares@umfst.ro (R.G.M.); alexandru.schiopu@med.lu.se (A.S.); 3 Department of Clinical Sciences Malmö, Lund University, 21428 Malmö, Sweden

**Keywords:** S100A9, myocardial infarction, apoptosis, hypertrophy, cardiac repair

## Abstract

Prognosis after myocardial infarction (MI) varies greatly depending on the extent of damaged area and the management of biological processes during recovery. Reportedly, the inhibition of the pro-inflammatory S100A9 reduces myocardial damage after MI. We hypothesize that a S100A9 blockade induces changes of major signaling pathways implicated in post-MI healing. Mass spectrometry-based proteomics and gene analyses of infarcted mice left ventricle were performed. The S100A9 blocker (ABR-23890) was given for 3 days after coronary ligation. At 3 and 7 days post-MI, ventricle samples were analyzed versus control and Sham-operated mice. Blockade of S100A9 modulated the expressed proteins involved in five biological processes: *leukocyte cell–cell adhesion*, *regulation of the muscle cell apoptotic process*, *regulation of the intrinsic apoptotic signaling pathway*, *sarcomere organization* and *cardiac muscle hypertrophy*. The blocker induced regulation of 36 proteins interacting with or targeted by the cellular tumor antigen p53, prevented myocardial compensatory hypertrophy, and reduced cardiac markers of post-ischemic stress. The blockade effect was prominent at day 7 post-MI when the quantitative features of the ventricle proteome were closer to controls. Blockade of S100A9 restores key biological processes altered post-MI. These processes could be valuable new pharmacological targets for the treatment of ischemic heart. Mass spectrometry data are available via ProteomeXchange with identifier PXD033683.

## 1. Introduction

Cardiac repair post-MI involves an early inflammatory phase, within 72 h, and a late proliferative phase, beyond 72 h [1]. The early phase involves expansion of the infarcted zone due to degradation of the inter-cardiomyocyte collagen by serine proteases and matrix metalloproteinases (MMPs). These enzymes are mainly released from neutrophils, the predominant leukocyte population infiltrating the myocardium within 24 h post-MI [2]. Neutrophils contribute to the clearance of necrotic cardiomyocytes and cellular debris and secrete mediators that promote further leucocyte recruitment and inflammation [3]. This initial phase is followed by the recruitment and activation of circulating monocytes, which dominate the second wave of myocardial myeloid cell recruitment, peaking around day 3 post-MI [4]. The infiltrating monocytes turn into inflammatory and reparatory macrophages in the tissue. Phagocytic clearance of apoptotic neutrophils and cardiomyocytes reprograms macrophages toward an anti-inflammatory and reparatory phenotype that dominates the late remodeling phase [4]. Following necrotic cell clearance and collagen synthesis, the infarcted area elongates and thins, leading to left ventricular dilation [1,2]. This late ventricular remodeling comprises changes at the cellular and molecular level, involving oxidative stress and apoptosis, myocyte hypertrophy, neurohormonal responses, ventricular dilation, and scar formation, among others [5].

Interventions that interfere with the cardiac wound healing process may promote adverse ventricular remodeling [2], leading to a worse patient prognosis. However, targeting the early inflammatory phase post-MI has previously shown important therapeutic potential. S100A9 and its dimerization partner S100A8 are pro-inflammatory mediators predominantly secreted by neutrophils that increase rapidly in the blood and in the heart of MI patients [6]. S100A8/A9 binds TLR4 and RAGE on myeloid and endothelial cells, promoting leukocyte recruitment and pro-inflammatory activation. Recently, Marinkovic et al. [7] have shown that a short-term S100A9 blockade during the peak inflammatory phase of the immune response limits and reduces local myocardial damage. We hypothesize that inhibition of S100A9 is a key factor that induces changes in major biological processes implicated in cardiac recovery post-MI, such as leukocyte adhesion and apoptosis. In view of the potential development of the S100A9 blockade as an immunomodulatory therapy in MI, it is essential to uncover its effects on the pathways involved in post-ischemic cardiac remodeling.

To address this important question, we performed mass spectrometry analyses of the left ventricular proteome during the inflammatory and reparative phase, at 3 and 7 days post-MI, in mice with permanent myocardial ischemia induced by coronary artery ligation. The most important differences in protein level between the groups were further validated by Western blot and correlated with the gene expression profile in the myocardium. We report that short-term pharmacological inhibition of S100A9 favorably modulates the presence and gene expression of numerous proteins involved in biological pathways mediating cardiac repair and remodeling post-MI. Further, we have identified the involvement of several previously undisclosed proteins as determinant contributors in these processes.

## 2. Results

### 2.1. Identification of Differentially Expressed Proteins (DEPs) in the Infarcted Left Ventricle by Mass Spectrometry

**Figure 1:**
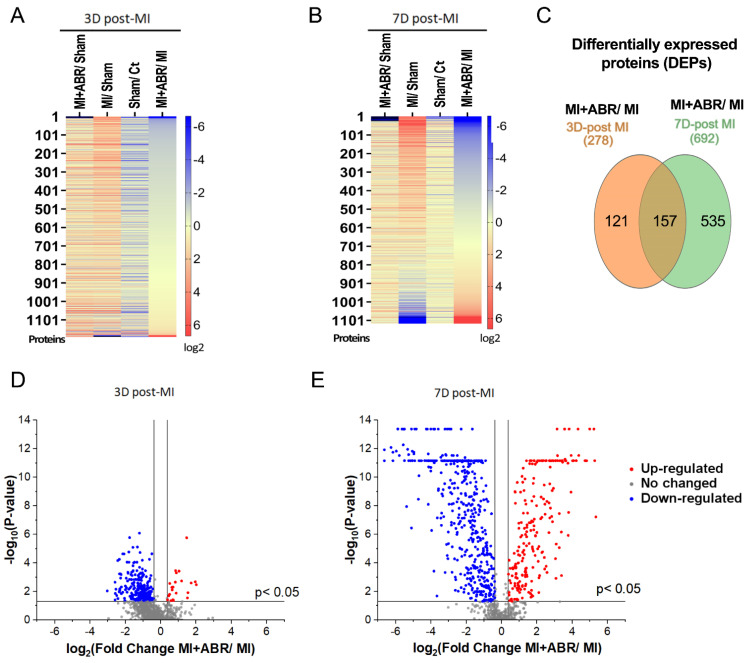
Proteomic analysis of left ventricle on day 3 and 7 post-MI. The left ventricle was separated from the heart, harvested and processed for future analysis. (**A**) At 3 days (3D) post-MI, 1192 proteins were identified in the unoperated control (Ct), Sham, MI, and MI + ABR groups. The relative protein expression is presented as a pairwise comparison between the groups, where deeper blue represents lower abundance and deeper red represents higher abundance. (**B**) At day 7 post-MI (7D), a total of 1117 proteins were identified. (**C**) Venn diagram showing the number of common and different up- or downregulated proteins in MI + ABR versus MI control hearts at 3 and 7 days post-MI (**D**,**E**). Volcano plots of differential protein abundance, with fold difference between the log2 normalized abundance in the MI + ABR and MI groups plotted versus −log10 adjusted *p* value, at 3 D post-MI and 7 D post-MI. Each dot represents a protein. Colored dots represent the expression profiles of significantly different proteins with a relative fold change of ≥1.30 or ≤0.77. The calculated *p* value was obtained using ANOVA hypothesis test and was adjusted using Benjamini-Hochberg correction for the false discovery rate.

In samples collected at day 3 post-MI, we identified 1192 quantifiable proteins with a Sequest Score ≥ 10 and unique peptide matches ≥ 1 (Figure 1A). At day 7 post-MI, we found 1117 common quantifiable proteins for all four experimental groups (Figure 1B). A Venn diagram was employed to illustrate the overlap of identified proteins from 3 and 7 days post-MI, limited to proteins that differed significantly between the MI + ABR group and the MI group (fold change > 1.30 or fold change < 0.77, *p* < 0.05). A total of 157 proteins were found to be differentially expressed between the two groups at both time points (Figure 1C). The number of differentially expressed proteins increased from day 3 to day 7, suggesting that the influence of the S100A9 blockade on protein expression in the myocardium extends beyond the 3-day treatment period. This is in line with previously published data showing that the short-term S100A9 blockade has long-term beneficial effects, leading to a functional gain in left ventricle (LV) function that gradually increases with time [8]. To further verify this data, we performed a volcano analysis of the normalized datasets pertaining to the two time points. The analysis on day 3 post-MI revealed 22 upregulated proteins (fold change > 1.30; *p* < 0.05) and 256 downregulated proteins (fold change < 0.77; *p* < 0.05) when comparing MI + ABR group to MI (Figure 1D). Conversely, the volcano plot specific for the day 7 analysis highlighted not only a greater number of differentially abundant proteins, but also a higher significance of regulation with 223 proteins being upregulated (fold change ≥ 1.30, *p* < 0.05) and 469 downregulated (fold change < 0.77; *p* < 0.05), (Figure 1E).

### 2.2. DEP Gene Ontology Analysis Revealed the Significant Enrichment of Key Cellular Processes Involved in Post-MI Recovery

**Figure 2:**
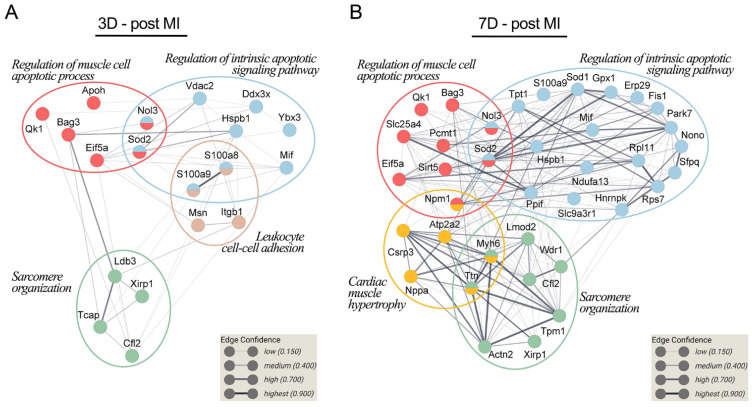
Associations between the biological processes related to ventricle repair and remodeling and differentially expressed proteins (DEPs) between the treatment groups. The protein–protein interaction networks at 3 days (**A**) and 7 days post-MI (**B**) were built using STRING 11.0. The proteins, identified by gene name, are represented as nodes. The thickness of the edge shows the strength of the interaction between two proteins.

To determine the biological processes related to post-MI ventricular remodeling that are regulated by the S100A9 blockade, all DEPs for each time point were input into STRING (version 11.0). Bioinformatic analysis of the Gene Ontology Biological Process (GO BP) demonstrated enrichment of the regulation of the muscle cell apoptotic process, regulation of the intrinsic apoptotic signaling pathway and sarcomere organization at both day 3 and 7 post-MI. In addition, leukocyte cell–cell adhesion was significantly enriched on day 3 post-MI (Figure 2A, brown circle), while cardiac muscle hypertrophy was significantly enriched on day 7 post-MI (Figure 2B, yellow circle). According to the GO analysis, only 46 proteins out of the total DEPs analyzed at the two time points were detected as being involved in the aforementioned biological processes. Figure 2 shows the relationship between these biological processes and the 46 proteins found to be differentially expressed after MI.

The 46 DEPs were manually curated and verified, and the applied confidence filters were well above the minimal thresholds (Table 1).

### 2.3. S100A9 Blockade Downregulates the Expression of Proteins Involved in Leukocyte Cell–Cell Adhesion during the Inflammatory Phase Post-MI

**Figure 3:**
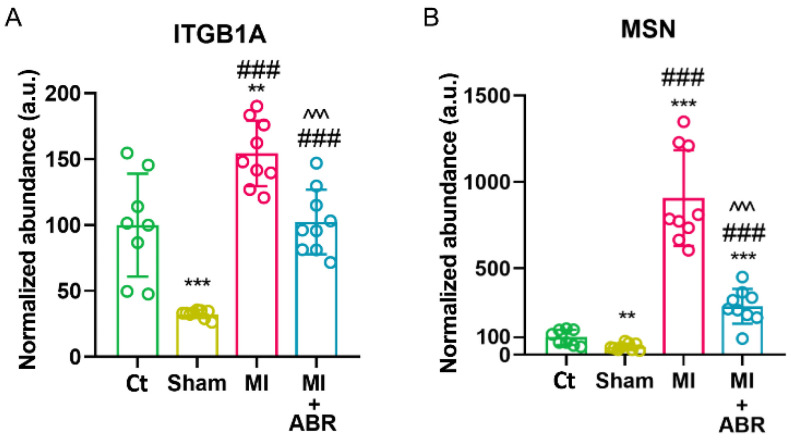
S100A9 blockade lowers the level of leukocyte cell–cell adhesion molecules in the infarcted myocardium on day 3 post-MI. Normalized spectral abundance of (**A**) ITGβ1A and (**B**) MSN in the left ventricle for controls (Ct, Sham), PBS-treated (MI) and ABR-treated (MI + ABR) infarcted mice. Data are presented as mean ± SD; ITGβ1A, integrin beta-1 isoform A; MSN, moesin; ** *p* < 0.01, *** *p* < 0.001 vs. Ct; ### *p* < 0.001 vs. Sham; ^^^ *p* < 0.001 vs. MI; *p* values were calculated using Student’s *t* test.

Leukocyte recruitment is regulated by a series of cellular and molecular events involving tethering, rolling, firm adhesion and trans-endothelial migration [9]. On day 3 post-MI, the cell adhesion molecule integrin beta-1 isoform A (ITGβ1A) was significantly increased in the MI group compared to unoperated controls (Ct) and Sham by 1.5-fold and 4.7-fold, respectively (Figure 3A). S100A9 blockade significantly decreased the abundance of ITGβ1A by ∼1.7-fold (*p* < 0.01) compared to the MI group (Figure 3A). In contrast, the isoform D of integrin β1, which is only expressed in cardiomyocytes, was not significantly altered between the groups. Moesin (MSN), the most abundant member of the ERM (Ezrin/radixin/moesin) protein family in leukocytes, mediates cellular shape changes, polarity and adhesion [10]. The abundance of moesin was significantly upregulated in the MI group compared to unoperated controls (Ct) and Sham by 9-fold and 19-fold, respectively (Figure 3B). Similar to ITGβ1A, moesin was significantly decreased by 3.2-fold (*p* < 0.01) in the LV of mice with MI treated with ABR-238901 compared to the MI group (Figure 3B). These results suggest that S100A9 stimulates leukocyte adhesion and recruitment post-MI and partially explain the previously reported inhibition of leukocyte recruitment in the infarcted myocardium induced by S100A9 blockade [8].

### 2.4. S100A9 Blockade Modulates the Apoptotic Process and Sarcomere Organization in Cardiomyocytes

**Figure 4:**
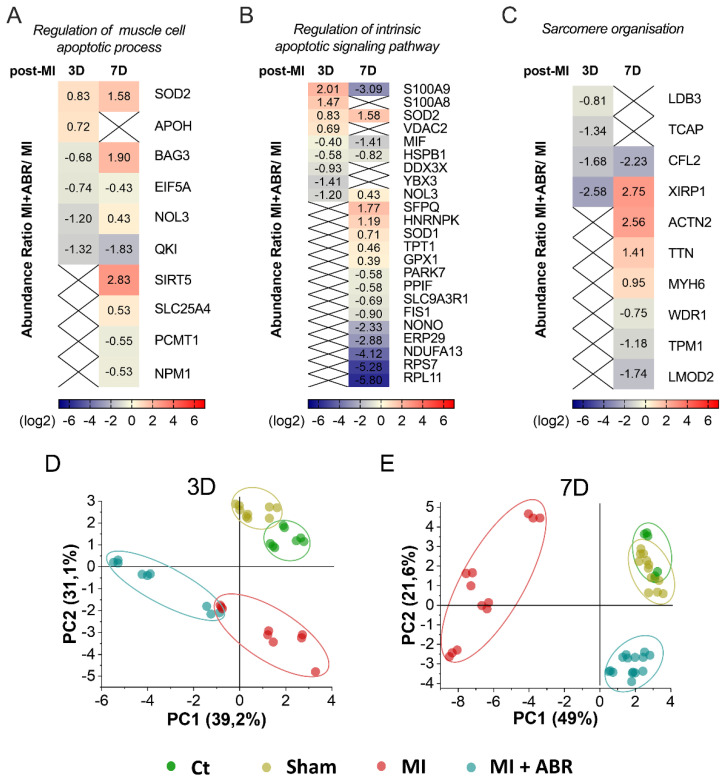
Heatmaps and principal component analysis (PCA) of the relative abundance of DEPs controlling biological processes involved in myocardial repair and remodeling. (**A**) DEPs involved in the regulation of the muscle cell apoptotic process, (**B**) regulation of the intrinsic apoptotic signaling pathway and (**C**) sarcomere organization. Red represents upregulated and blue represents downregulated proteins in the LV collected from the MI + ABR group compared to MI mice. DEPs were considered to be significant at an adjusted *p* value < 0.05. The calculated *p* value was obtained using ANOVA hypothesis test and adjusted using Benjamini-Hochberg correction for the false discovery rate (FDR). Colored bars indicate the expression levels. X-marking represents proteins that were not significantly different between the groups. PCA score plot based on the LC-MS/MS data of DEPs involved in the regulation of the muscle cell apoptotic process, regulation of the intrinsic apoptotic signaling pathway, and sarcomere organization GO BP for all experimental groups at 3 days (**D**) and 7 days (**E**) post-MI. An ellipse highlights the data points coming from one group samples, which were run in three technical replicates (dots) and are added for visual purposes only. The colors of dots are: green, control (Ct) unoperated mice; dark yellow, Sham operated mice; red, MI mice; blue, MI + ABR mice. All protein short names are mentioned by the gene nomenclature guidelines.

Apoptosis, through the extrinsic (death receptor) and intrinsic (mitochondrial) pathways, is a major determinant of myocardial loss after acute MI and contributes to left ventricular remodeling and development of heart failure [11]. We found that several proteins associated with the apoptotic processes were significantly altered in the MI groups at both 3 and 7 days post-MI, involved mainly in the regulation of the muscle cell apoptotic process and the regulation of the intrinsic apoptotic signaling pathway. The GO BP enrichment analysis of DEPs at 3 days post-MI identified six proteins involved in the regulation of the muscle cell apoptotic process (FDR = 0.0043). In MI mice treated with ABR-238901, the mitochondrial superoxide dismutase (SOD2) and the beta-2-glycoprotein 1 (APOH) were upregulated, while the BAG family molecular chaperone regulator 3 (BAG3), eukaryotic translation initiation factor 5A-1 (EIF5A), nucleolar protein 3 (NOL3), protein quaking (QKI) were downregulated relatively to the MI control group (Figure 4A). At 7 days post-MI, we detected nine DEPs associated with the regulation of the muscle cell apoptotic process (FDR = 0.0073). SOD2, BAG3, NOL3, NAD-dependent protein deacylase sirtuin-5 mitochondrial (SIRT5), and ADP/ATP translocase 1 (SLC25A4) were upregulated by the treatment, while EIF5A, QKI, protein-L-isoaspartate (D-aspartate) O-methyltransferase (PCMT1), and nucleophosmin (NPM1) were downregulated in the LV of mice with MI treated with ABR-238901 compared to the MI group (Figure 4A).

At 3 days post-MI, we detected nine DEPs linked to the regulation of the intrinsic apoptotic signaling pathway (FDR = 0.006). Among these proteins, S100A9, S100A8, SOD2 and the voltage-dependent anion-selective channel protein 2 (VDAC2) were upregulated, while macrophage migration inhibitory factor (MIF), heat shock protein beta-1 (HSPB1), ATP-dependent RNA helicase DDX3X (DDX3X), Y-box-binding protein 3 (YBX3) and NOL3 were downregulated by the S100A9 blockade (Figure 4B). At 7 days post-MI, the analysis highlighted 19 DEPs involved in the regulation of the intrinsic apoptotic signaling pathway (FDR = 4.80 × 10^−5^), (Figure 4B). Compared to the MI group, in the MI + ABR group, we found 7 upregulated and 12 downregulated proteins. The upregulated proteins were SOD2, NOL3, splicing factor proline- and glutamine-rich (SFPQ), heterogeneous nuclear ribonucleoprotein K (HNRNPK), superoxide dismutase [Cu-Zn] (SOD1), translationally-controlled tumor protein (TPT1), and glutathione peroxidase 1 (GPX1). In contrast, the S100A9 blockade led to a decline in myocardial abundance of S100A9, MIF, HSPB1, protein/nucleic acid deglycase DJ-1 (PARK7), peptidyl-prolyl cis-trans isomerase F mitochondrial (PPIF), Na(+)/H(+) exchange regulatory cofactor NHE-RF1 (SLC9A3R1), mitochondrial fission 1 protein (FIS1), non-POU domain-containing octamer-binding protein (NONO), endoplasmic reticulum resident protein 29 (ERP29), NADH dehydrogenase [ubiquinone] 1 alpha subcomplex subunit 13 (NDUFA13), 40S ribosomal protein S7 (RPS7), and 60S ribosomal protein L11 (RPL11).

LIM domain-binding protein 3 (LDB3), telethonin (TCAP), cofilin-2 (CFL2) and xin actin-binding repeat-containing protein 1 (XIRP1), proteins involved in sarcomere organisation (FDR = 0.0033) exhibited lower abundance in the LV of MI mice treated with ABR-238901 compared to MI on day 3 post-MI. On day 7 post-MI, we found eight DEPs associated with sarcomere organization (FDR = 0.00011). XIRP1, alpha-actinin-2 (ACTN2), titin (TTN) and myosin-6 (MYH6) were upregulated, and CFL2, WD repeat-containing protein 1 (WDR1), tropomyosin alpha-1 chain (TPM1) and leiomodin-2 (LMOD2) were downregulated in the MI + ABR group versus the MI group (Figure 4C).

To better highlight the effect of S100A9 inhibition at day 3 and 7 post-MI on the regulation of the muscle cell apoptotic process, the intrinsic apoptotic signaling pathway and sarcomere organization, we applied principal component-based multivariate statistics (PCA). The analysis performed on day 3 data did not reveal a clear differentiation of the proteome alterations between the MI and the MI + ABR groups (Figure 4D). However, PCA performed on day 7 post-MI samples revealed prominent differences among the quantitative features of the two groups, demonstrating that the protein content in LV from mice receiving ABR treatment was markedly closer to the two control groups (Ct, Sham) than to the MI animals (Figure 4E), which demonstrated a clear distancing and separation from all other groups.

### 2.5. S100A9 Blockade Regulates Cardiac Hypertrophy Starting at Day 7 Post-MI

**Figure 5:**
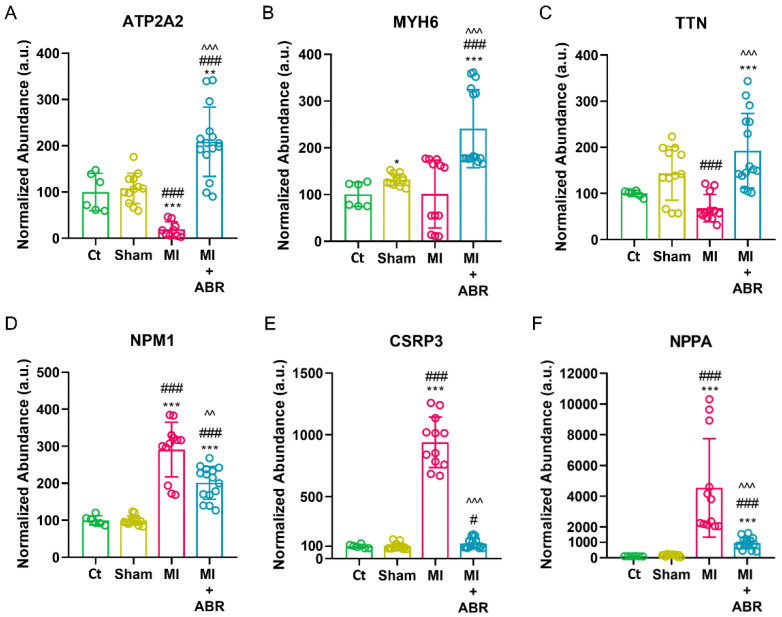
Impact of 3-day S100A9 inhibition on proteins involved in cardiac hypertrophy. LC MS/MS analysis showing the abundance of ATP2A2 (**A**), MYH6 (**B**), TTN (**C**), NPM1 (**D**), CSRP3 (**E**), and NPPA (**F**) in the experimental groups at day 7 post-MI. Data are presented as mean ± SD. Each sample was run in triplicate; * *p* < 0.05, ** *p* < 0.01, *** *p* < 0.001 vs. Ct; # *p* < 0.05, ### *p* < 0.001 vs. Sham; ^^ *p* < 0.01, ^^^ *p* < 0.001 vs. MI; *p* values were calculated using Student’s *t* test; ATP2A2, ATPase 2; MYH6, myosin-6; TTN, titin; NPM1, nucleophosmin; CSRP3, glycine-rich protein 3; NPPA, natriuretic peptide A.

Hypertrophy of the remote myocardium post-MI occurs as a compensatory mechanism for the lost muscle mass. However, excessive LV hypertrophy and cardiac remodeling are associated with an adverse outcome [12]. At 3 days post-MI, there was no significant difference between the treated groups regarding the abundance of proteins involved in cardiac muscle hypertrophy according to GO BP. However, at 7 days post-MI, the S100A9 blockade significantly influenced the levels of six proteins associated with these processes (Figure 5).

The abundance of the sarcoplasmic/endoplasmic reticulum calcium ATPase 2 (ATP2A2) and myosin-6 (MYH6) were significantly increased in the MI + ABR group compared to unoperated controls, Sham and MI groups (Figure 5A,B). Titin (TTN) was also significantly higher in MI + ABR mice compared to unoperated and MI mice (Figure 5C). In contrast, nucleophosmin (NPM1), glycine-rich protein 3 (CSRP3) and natriuretic peptide A (NPPA) were lowered by the ABR-238901 treatment (Figure 5D–F).

### 2.6. The Short-Term S100A9 Blockade Significantly Modulates the Cellular Tumor Antigen p53

It has been previously shown that the cellular tumor antigen p53 (p53), besides its regulatory role of the extrinsic and intrinsic apoptotic pathways, also acts as a pleiotropic regulator of cardiac structure and function [13]. We examined the effect of S100A9 inhibition at 3 and 7 days post-MI on the mRNA and protein expression of p53 in the infarcted ventricle.

**Figure 6:**
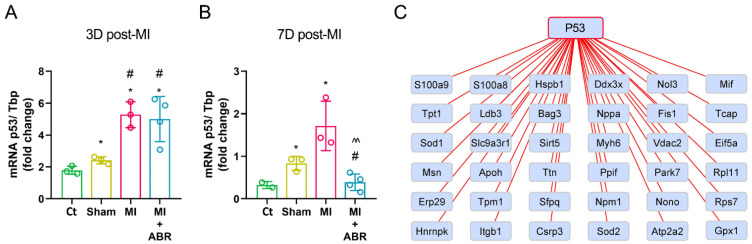
Effect of S100A9 blockade on p53 in the LV post-MI. mRNA expression levels of cellular tumor antigen P53 (P53) in the LV of unoperated control Ct, Sham, MI and MI + ABR groups quantified by real-time PCR using Tbp (TATA sequence binding protein) as internal control, at 3 (**A**) and 7 (**B**) days post-MI. Interactions of p53 with DEPs implicated in leukocyte cell–cell adhesion, regulation of the muscle cell apoptotic process, regulation of the intrinsic apoptotic signaling pathway, sarcomere organization and cardiac muscle hypertrophy GO biological processes (**C**). The proteins (identified by gene name) are represented as rectangle nodes. Data are presented as mean ± SD; * *p* < 0.05 vs. Ct; # *p* < 0.05 vs. Sham; ^^ *p* < 0.01 vs. MI; *p* values were calculated using Student’s *t* test.

We found that both MI groups had a significantly increased p53 gene expression by ∼2.9-fold at 3 days post-MI (Figure 6A). At 7-days, the p53 gene expression was downregulated by 3.67-fold in the MI + ABR group compared to the MI group treated with PBS (Figure 6B).

Next, we searched for DEPs that interact with p53. Based on the information obtained from the STRING database, 36 out of 46 DEPs linked to the GO BP examined before were detected to interact with p53 (Figure 6C). These results suggest that p53 plays a central role in post-MI LV repair and remodeling, and its expression is modulated by the S100A9 blockade.

### 2.7. Western Blot Validation of the LC-MS/MS Data

**Figure 7:**
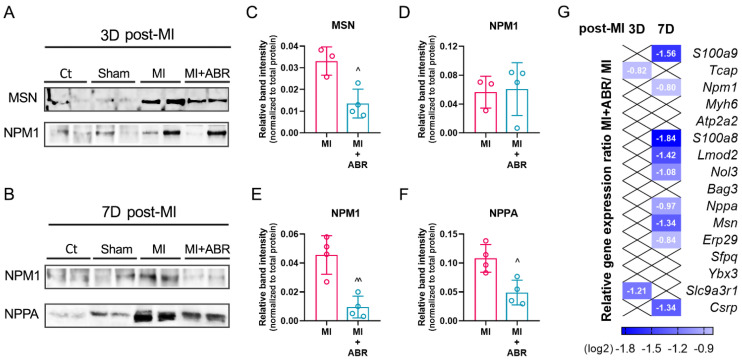
Validation of several proteome data in the LV by Western blot analysis and qPCR. Representative Western blots for all experimental groups at 3 and 7 days post-MI (**A**,**B**). Semi-quantitative analyses displaying the protein levels of MSN and NPM1 normalized to total protein for MI and MI + ABR groups at 3 days post-MI (**C**,**D**). Semi-quantitative analyses showing protein levels of NPM1 and NPPA normalized to total protein for MI and MI + ABR groups at 7 days post-MI (**E**,**F**). Heatmap profile of differentially expressed genes at 3 and 7 days post-MI showing mRNA expression levels of Msn, S100a9, S100a8, Npm1, Sfpq, Slc9a3r1, Erp29, Nppa, Bag3, Nol3, Lmod2, Myh6, Atp2a2, Tcap, Ybx3 and Csrp3 in the left ventricle of MI and MI + ABR groups were quantified by real-time PCR, using Tbp as internal control (**G**). The gene expression data are presented as shades of blue for the log2 ratio of gene expression in MI + ABR vs. MI, depicting different levels of downregulation. Only the ratios for genes with significant differential expression are shown (*p* < 0.05). The Western blot data represent the mean ± SD; ^ *p* < 0.05, ^^ *p* < 0.01 vs. MI using Student’s *t* test.

We performed Western blot assays to confirm the differences found for three DEPs associated with leukocyte cell–cell adhesion, muscle cell apoptosis and cardiac hypertrophy in the infarcted LV at 3 and 7 days post-MI (Figure 7A,B). The analysis confirmed that the level of MSN (Figure 7C) was three-fold lower (*p* < 0.05) in the LV of mice with MI treated with ABR-238901 compared to the MI mice at 3 days post-MI.

The levels of NPM1 were similar in the two treatment groups at 3 days post-MI (Figure 7D), but were decreased in LV samples collected 7 days post-MI (*p* < 0.05) (Figure 7E). Similarly, the S100A9 blockade significantly reduced the levels of NPPA (*p* < 0.05) compared with the MI group on day 7 after MI (Figure 7F). These results are in accordance with the LC−MS/MS proteomics data (Figure 3B, Figure 4A and Figure 5F).

### 2.8. Gene Expression Analysis of Differentially Expressed Proteins (DEPs)

Lastly, 16 selected genes encoding for the identified DEPs were analyzed by RT-PCR. The gene selection criterion consisted of choosing at least three genes encoding common DEPs (S100a8, S100a9, Mhy6, Npm1, Nol3) as well as specific (Msn, Sfpq, Slc9a3r1, Erp29, Nppa, Bag3, Lmod2, Myh6, Atp2a2, Tcap, Ybx3, Csrp3) to the biological processes studied previously. At 3 days post-MI, compared to the Sham group, in the MI group, we detected significantly higher gene expression for Msn, S100a9, S100a8, Npm1, Sfpq, Slc9a3r1, Erp29, and Nppa, while the expression of Bag3, Nol3, Lmod2, Myh6, Atp2a2 were significantly downregulated (Figure S1 in the Supplementary Material). The S100A9 blockade significantly reduced the expression of Slc9a3r1 (2.3-fold) and Tcap (1.78-fold) compared to the MI controls treated with PBS (Figure 7G).

At 7 days post-MI, the gene expression analysis demonstrated that the Msn, S100a9, S100a8, Npm1, Sfpq, Slc9a3r1, Erp29, Nppa, Ybx3, Nol3 and Lmod2 genes were significantly upregulated, while Atp2a2, Tcap and Myh6 were downregulated in the MI versus the Sham group (Figure S2 in the Supplementary Material). The anti-S100A9 treatment led to significantly downregulated gene expression for Msn (2.5-fold), S100a9 (2.9-fold), S100a8 (3.6-fold), Npm1 (1.7-fold), Erp29 (1.78-fold), Nppa (1.9-fold), Nol3 (2.1-fold), Lmod2 (2.6-fold), and Csrp3 (2.5-fold) compared to the MI group (Figure 7G).

## 3. Discussion

Cardiac repair and remodeling after MI involve changes at the cellular and molecular level that affect ventricular size, morphology, and function [14]. Previous proteomic studies have highlighted important changes in the cardiac proteome after MI [15,16,17,18]. In a previous study, we have shown that a short-term S100A9 blockade inhibits inflammation, reduces myocardial damage and leads to a long-term gain in cardiac function post-MI [8]. Here, we present data on the impact of S100A9 inhibition on protein levels and biological processes involved in post-MI ventricular repair and remodeling. We performed a proteomic analysis of LV extracts during the inflammatory (3 days) and reparatory phase (7 days) post-MI and performed a bioinformatic analysis based on the mass spectrometry data linked to gene expression. The results reveal important effects of S100A9 inhibition on differential expressed proteins involved in five biological processes according to GO: leukocyte cell–cell adhesion, regulation of the muscle cell apoptotic process, regulation of the intrinsic apoptotic signaling pathway, sarcomere organization and cardiac muscle hypertrophy.

Previous studies have reported that the extracellular S100A8/A9 enhances immune cell infiltration into the infarcted myocardium by upregulating integrin Mac-1 expression [19] and by binding to endothelial surface proteoglycans via the S100A9 subunit [20]. In the present study, we found an increased abundance of ITGB1A in the infarcted myocardium compared with non-infarcted controls during the inflammatory phase, which is in line with previous results demonstrating that inflammatory cells contribute to ITGB1A production [21]. We also show for the first time that the protein moesin is increased in the infarcted LV at 3 days post-MI compared to non-infarcted controls. Panicker et al. have previously demonstrated that neutrophil signaling initiated through P-selectin glycoprotein ligand-1 requires moesin to activate ERKs and release neutrophil extracellular traps and that moesin-deficient neutrophils have defective integrin outside-in signaling and reduced adhesion strength [22]. Moesin also promotes monocyte transendothelial migration [23] and was found to be upregulated in cardiomyocytes isolated from hearts with experimental autoimmune myocarditis [24]. The S100A9 blockade with ABR-238901 reduced the abundance of ITGB1A and moesin in the LV during the inflammatory phase, which potentially contributes to the immunomodulatory effects of the compound.

S100A8/A9 has been found to directly induce cardiomyocyte death in the early stage of MI by suppressing mitochondrial function [6]. Conversely, mice treated with ABR-238901 had a reduced percentage of apoptotic cells in the myocardium at 3 days post-MI [8]. The tumor suppressor p53 is an important regulator of the cardiac transcriptome under physiological conditions and a master promoter of apoptosis in response to acute stress [13]. Our current data demonstrate that the S100A9 blockade interferes with the apoptotic process by modulating 36 proteins that interact with or are targeted by p53. These findings are in line with a previous report demonstrating an important role of S100A9 as a downstream mediator of p53-dependent cellular apoptosis [25]. We show that the S100A9 blockade upregulates several proteins with anti-apoptotic properties and downregulates a number of pro-apoptotic factors involved in signaling pathways related to p53. Although the treatment was discontinued on day 3, we found clear evidence of its anti-apoptotic effects on day 7. The data support the long-lasting protective effects of the treatment and extend the previous study of Marinković et al., demonstrating progressive functional gain in ABR-238901-treated mice compared to controls [8].

The proteins with roles in the regulation of the muscle cell apoptotic process that were highly upregulated on day 7 are SOD2, BAG3 and SIRT5. These proteins have pleiotropic cardioprotective effects, including inhibition of pro-apoptotic pathways [26,27], reducing cardiac oxidative stress [28,29], promoting autophagy [30] and normalization of myocyte contractility [28,29,30]. The S100A9 blockade also increased cardiac levels of NOL3 and SLC25A4, albeit to a lower extent. NOL3 is a potent inhibitor of multiple apoptotic pathways with a post-ischemic cardioprotective effect [31]. In cardiomyocytes exposed to oxidative stress, p53 counteracts these protective effects by transcriptionally suppressing NOL3 production [32]. SLC25A4 is an effective protective factor in myocardial ischemia with an important role in keeping mitochondrial integrity and decreasing oxidative stress in infarcted heart tissue when it is overexpressed [33]. APOH, which was found to be upregulated on day 3 post-MI, mediates efficient recognition and removal of apoptotic cells by macrophages [34], accelerating the resolution of inflammation and preventing adverse remodeling.

The treatment lowered the muscle cell apoptotic regulators EIF5A, QKI, PCMT1 and NPM1. The downregulation of EIF5A, a known stimulator of p53 expression and transcriptional activity [35], is in line with the anti-apoptotic effects of the treatment discussed above. The RNA-binding protein QKI is a p53 target gene. The QKI-7 isoform of the protein induces apoptosis when localized in the cytoplasm, and its nuclear translocation prevents this effect [36]. The downregulation of PCMT1 and NPM1 is somewhat surprising. PCMT1 is an enzyme with methyltransferase activity, and its overexpression confers resistance to the hypoxia/reoxygenation-induced apoptosis in cardiac cells [37]. Overexpression of NPM1 is thought to suppress the translocation of p53 from the nucleus to the mitochondria, preventing p53-mediated apoptosis [38]. We found higher levels of the nucleolar protein NPM1 in the LV of mice with MI compared to non-infarcted controls, which is in line with previously published data [39]. The reduced expression of PCMT1 and NPM1 at 7 days post-MI in mice receiving the S100A9 blockade may be due to a reduced need for this cellular survival mechanism in the context of decreased cardiomyocyte apoptosis.

Our bioinformatic analysis showed that several DEPs were associated with the regulation of the intrinsic apoptotic signaling pathway. Among these proteins, the nucleolar proteins DDX3X, NONO, RPS7 and RPL11, as well as the mitochondrial fission protein FIS1 and MIF, were potently reduced by the S100A9 blockade. Nucleolar enlargement is indicative of increased protein synthesis and growth and is one of the early changes observed in hypertrophied human hearts [40]. FIS1 is enhanced after hypoxia/reperfusion injury and was previously associated with cardiomyocyte hypertrophy [41]. MIF levels are also lowered, probably as a consequence of the reduced presence and activation of inflammatory cells. In the setting of extended myocardial ischemia, reflected by our model of permanent coronary artery ligation, MIF has been found to be pro-inflammatory and to contribute to cardiac dysfunction and remodeling [42]. PPIF and NDUFA13 are mitochondrial inner membrane proteins, and our experiments revealed that the S100A9 blockade downregulated their respective abundance in the infarcted LV. Inhibition or genetic ablation of PPIF was found to confer resistance against myocardial ischemia/reperfusion injury and development of heart failure [43]. Similarly, cardiac-specific heterozygous NDUFA13 knockout mice have increased resistance to ischemia/reperfusion-induced cardiomyocyte apoptosis [44]. We also detected a low abundance of SLC9A3R and ERP29. The importance of these proteins in cardiac remodeling post-MI has not been investigated thus far.

We have found significant differences in the expression of the DNA- and RNA-binding proteins YBX3, SFPQ and HNRNPK between the experimental groups, and we are the first to report the upregulation of Ybx3 and Sfpq in the myocardium post-MI. The Ybx3 gene was found to be upregulated in peripheral blood cells from patients with MI compared to normal individuals [45]. This protein is an important regulator of amino acid levels in cells [46], but its roles in MI are thus far unknown. Loss of SFPQ in skeletal muscle causes progressive muscle mass reduction and disturbance of important metabolic pathways [47]. The S100A9 blockade increased cardiac levels of SFPQ, which might contribute to the protective effects. The treatment also increased the protein levels of HNRNPK in the heart. Swiatkowska et al. have shown that HNRNPK amplifies p53 binding to target genes under stress conditions and also promotes p53 expression [48]. The upregulation of HNRNPK levels in the heart of mice receiving the S100A9 blockade might represent a compensatory mechanism attempting to re-establish the p53 signaling.

Other DEPs involved in the regulation of the intrinsic apoptotic signaling pathway that were upregulated are SOD1, GPX1, VDAC2 and TPT1. Increased expression of SOD1 in cardiomyocytes has previously been shown to confer protection against post-ischemic injury [49]. GPX1 is a peroxidase with a protective role against oxidative damage that has been shown to reduce post-MI ventricular dilation and dysfunction, as well as myocyte hypertrophy, apoptosis, and interstitial fibrosis in the non-infarcted myocardium [50]. VDAC2 plays important roles in mitochondrial communication with other cellular components and in bioenergetic metabolism, and has been shown to attenuate cell death mechanisms [51]. Similarly, the overexpression of the TPT1 gene has been found to protect against doxorubicin-induced cardiomyocyte death [52].

On day 7 post-MI, the abundance of XIRP1, ACTN2, TTN, and MYH6 was significantly higher in ABR-238901-treated mice. All these proteins participate in the physiological organization and function of the sarcomere, playing central roles in cardiac elasticity and contractility. The higher abundance of these proteins in the LV witnesses the efficiency of the treatment in preserving cardiac structure and is likely to directly contribute to the higher cardiac systolic function in mice receiving the S100A9 blockade [8]. Conversely, the level of CFL2, WDR1, TPM1 and LMOD2 was downregulated. CFL2 belongs to a family of actin-remodeling proteins and plays an important role in normal muscle function [53]. WDR1 is a cofilin (CFL) co-factor that amplifies actin filament fragmentation [54]. CFL2 suppression has been found to have a protective effect against the development of post-ischemic heart failure in mice [55]. Consequently, the lower levels of CFL2 and WDR1 in LV of MI mice treated with ABR-238901 might help preserve the structural integrity of actin and improve function. TPM1 regulates the actin–myosin interaction, with important roles in cardiac muscle relaxation and contraction, and mutations in the Tpm1 gene lead to both hypertrophic and dilated cardiomyopathy [56]. As TPM1 acts as an inhibitor of the actin–myosin interaction, lower levels of the protein might be part of a temporary compensatory mechanism that favors cardiomyocyte contraction. However, this hypothesis is speculative, as the post-ischemic changes in TPM1 expression and function are poorly understood. LMOD2 mediates actin polymerization and elongation of the thin actin filaments. Whereas LMOD2 is required for the normal sarcomere organization in the heart, cardiac-specific overexpression of Lmod2 led to elongated actin thin filaments, cardiac hypertrophy, interstitial fibrosis, and heart failure [57].

Our results showed that S100A9 inhibition impacted the abundance of proteins associated with cardiac muscle hypertrophy on late stages of post-MI. ATP2A2 is an enzyme involved in intracellular Ca2+ trafficking from the cytosol to the sarcoplasmic/endoplasmic reticulum, with a major role in excitation/contraction coupling. ATP2A2 inhibition causes Ca2+ overload and cardiomyocyte dysfunction [58], and gene therapies enhancing ATP2A2 expression have been proposed for the treatment of heart failure [59]. In our experiments, cardiac ATP2A2 abundance was highly upregulated by the treatment. CSRP3, also known as cardiac LIM protein, is a cytoskeleton-associated sensor of mechanical stress–strain. Overexpression, aberrations in oligomerization and nuclear accumulation of CSRP3 have been detected in animal models of MI and human heart failure and are thought to stimulate ribosomal protein synthesis in the nucleolus, promoting LV hypertrophy [60]. NPPA is synthesized by the atria in response to volume and pressure overload in heart failure, hypertension and MI, and it reduces blood pressure by inducing vasodilation and lowering plasma volume [61]. In our study, both proteins were significantly increased after MI and potently reduced by the S100A9 blockade, possibly witnessing reduced hemodynamic overload and mechanical stress.

Since both infarcted anterior wall and apical myocardium, as well as remote septal and back wall myocardium, have been collected for the analysis, we cannot exclude that technical variance in MI size between the animals might have affected the abundance of certain proteins. Moreover, in our previous paper [8], we have found that the S100A9 blockade slightly reduces the size of the MI on days 3 and 7.

## 4. Materials and Methods

### 4.1. Reagents

All reagents for liquid chromatography (LC) and mass spectrometry (MS) analysis were of LC-MS grade and purchased from Merck (Darmstadt, Germany). Trypsin Gold was purchased from Promega (Madison, WI, USA). C18 solid phase extraction columns were purchased from Waters Corporation (Milford, MA, USA). Millipore Protease Inhibitor Cocktail Set I was purchased from Merck (Darmstadt, Germany). Invitrogen SuperScript™ IV First-Strand Synthesis System and Applied Biosystems PowerUp SYBR Green PCR Master Mix were purchased from Thermo Fisher Scientific (Vilnius, Lithuania). Ambion TRIzol Reagent was purchased from Sigma-Aldrich (St. Louis, MO, USA). Invitrogen nucleophosmin monoclonal antibody (NA24), Invitrogen natriuretic peptides A polyclonal antibody, Pierce BCA Protein Assay Kit and Pierce ECL chemiluminescence kit were purchased from Thermo Scientific (Rockford, IL, USA). The moesin polyclonal antibody was purchased from Cell Signaling Technology (Danvers, MA, USA). The S100A8/A9 inhibitor (ABR-238901) was a gift from Active Biotech AB (Lund, Sweden).

### 4.2. Mouse Model of Myocardial Infarction

Eight- to twelve-week-old C57BL/6 mice (weighing 19–25 g) underwent permanent left coronary artery ligation according to a previously described method [62] to induce MI. Briefly, the mice were positioned on a heating pad to maintain their body temperature at 37 ± 0.5 °C and were anesthetized with 4% isoflurane using a continuous delivery system. The fur around the left chest area was removed using depilatory cream, and a small skin incision was made. Next, the heart was exposed through an opening at the fourth intercostal space, and the left anterior descending artery was ligated using a 6.0 silk suture. Subsequently, the heart was placed back into the thorax, the air was evacuated manually, and the skin was closed. The Sham group was submitted to the same surgical procedure, except that the ligature was not tied up and was removed. All mice were housed under controlled conditions of light (12 h light/dark cycle), temperature (22 ± 1 °C) and humidity (60 ± 5%) and free access to food and water. The animals were euthanized under general anesthesia induced by i.p. injection of a ketamine-xylazine (100/20 mg/kg body weight) solution.

All experimental procedures involving animals have been approved by the Ethics Committee of ICBP “N. Simionescu” and by the National Sanitary Veterinary and Food Safety Authority (no. 425/22.10.2018) in accordance with Directive 2010/63 of European Union. The study was conducted in accordance with the National Institutes of Health guide for the care and use of Laboratory animals (NIH Publications no. 8023, revised 1978), and Romanian Law no. 471/2002.

### 4.3. Experimental Design

**Figure 8:**
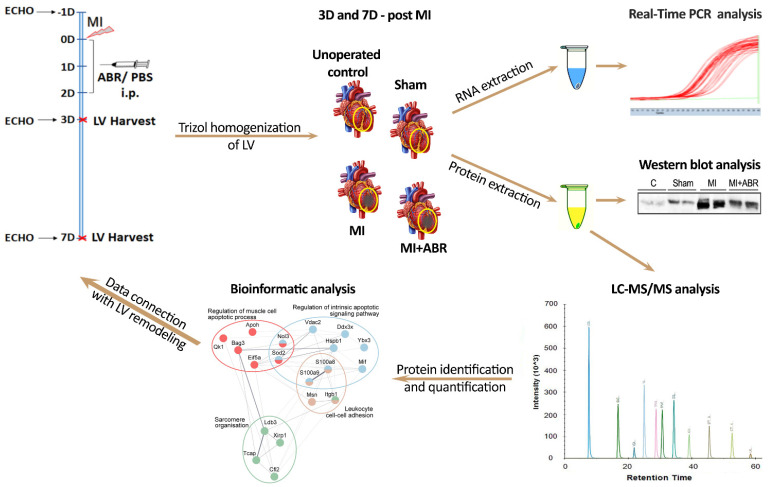
Schematic diagram of the experimental design. Mice were subjected to myocardial infarction (MI) by permanent left coronary artery ligation. The S100A9 inhibitor ABR-238901 (ABR, 30mg/kg diluted in PBS) or PBS were administered intraperitoneally (i.p.) immediately (0D), at 1 day (1D) and at 2 days (2D) after MI. Echocardiography (ECHO) was performed one day before MI (1D) and at 3 (3D) and 7 days (7D) after MI. The left ventricle of control mice (unoperated and Sham) and mice with induced infarction (MI and MI + ABR) were harvested from the indicated area (yellow circles) at 3 days and 7 days post-MI and homogenized with TRIzol Reagent for RNA and protein isolation. The RNA samples were analyzed by real-time PCR assays. The protein samples were analyzed by Western blot and LC-MS/MS, followed by bioinformatic analysis.

Initially, a total of 32 C57BL6 mice were included in the experiment. Twenty of them were subjected to permanent coronary ischemia surgical procedure, and each animal was examined by electrocardiography and echocardiography (ECHO) to confirm the MI. Four animals with left ventricular ejection fraction >40% calculated on parasternal long-axis (pLAX) view were excluded from the experimental group. The remaining mice for the 3 and 7 days post-MI experiments were 5 controls (Ct group), 7 Sham operated mice (Sham group), and 7 mice with MI, which were treated with PBS (MI group) and 9 mice with MI treated with 30 mg/kg ABR-238901 diluted in PBS (MI + ABR group). The experimental design is depicted in Figure 8.

### 4.4. RNA and Protein Extraction

At 3 and 7 days after MI, the hearts of unoperated control, Sham, MI and MI + ABR mice were harvested and washed with PBS containing a protease inhibitor cocktail. Next, the cardiac muscle below the ligature point of the left ventricle (LV) was removed, rapidly frozen with liquid nitrogen and preserved at −80 °C. RNA and proteins were extracted using TRIzol Reagent according to the manufacturer’s protocol. Briefly, LV tissue was homogenized in TRIzol Reagent using a Polytron PT 1300 D homogenizer (Kinematica, Lucerne, Switzerland), incubated with chloroform and centrifuged. The resulting upper aqueous phase was used for RNA precipitation, and the lower organic phase was used for protein precipitation. The protein pellet was solubilized in a lysis buffer containing 8 M urea, 1% sodium deoxycholate (DOC), 100 mM Tris-HCl (pH 7.5) and a protease inhibitor cocktail. Protein concentrations were determined by the BCA Protein Assay Kit. The purity and concentration of RNA was determined with a NanoDrop Lite spectrophotometer (Thermo Scientific). Only the RNA samples with 260/280 absorption ratio between 1.8 and 2.1 were used for further analysis.

### 4.5. Liquid Chromatography Coupled to Mass Spectrometry (LC-MS/MS) Analysis

First, 50 µg of proteins from each sample was purified by acetone precipitation, reduced with DTT (20 mM) in a buffer containing 8 M urea, 0.1 M Tris-HCl (pH 8.8) and 0.1 mM EDTA, and alkylated using 80 mM iodoacetamide in 0.1 M Tris-HCl and 0.1 mM EDTA buffer, in the dark, under agitation. Samples were proteolyzed overnight at 37 °C using Trypsin Gold (1:20 *w*/*w*), and formic acid was added to the resulting peptide mixtures to pH 2.5, in order to inhibit the enzymatic activity and promote DOC precipitation. The resulted peptides were desalted on Sep-Pak C18 columns (Waters) and concentrated with the Concentrator plus system (Eppendorf, Hamburg, Germany).

The LC-MS/MS analysis was conducted using the Easy nLC II liquid chromatograph (Thermo Fisher Scientific, Waltham, MA, USA) coupled to the LTQ-Velos Orbitrap mass spectrometer (Thermo Fisher Scientific). The peptides were loaded on a trap column (Thermo Scientific Easy Column—2 cm length, 100 μm inner diameter, 5 μm particle size, 100 Å pore size) and separated on the 10 cm EASY column (Thermo Scientific—75 μm inner diameter, 3 μm particle size, 100 Å pore size) using a 300 nl/min 3–25% solvent B gradient (0.1% formic acid in acetonitrile, while solvent A was 0.1% formic acid in water) over 120 min. The spectra of the separated peptides were acquired by the mass spectrometer, which was operated in a top 12 data-dependent configuration at 60 k resolving power for a full scan across the 350–1700 *m*/*z* domain. Collision-induced dissociation (CID) was enabled for parent ion fragmentation. To reduce the effect of experimental variation, each sample was injected in triplicate.

### 4.6. Data Processing

The acquired raw data were analyzed with the Proteome Discoverer 2.4 software (Thermo Fisher Scientific). The protein inference was performed using the UniProtKB/Swiss-Prot mouse reference protein database (UP000000589 Proteome ID, v.04.2019) using the following parameters. Enzyme: trypsin; maximum miss cleavages: 2; fixed modification: cysteine carbamidomethylation; variable modifications: oxidation of methionine and deamidation of asparagine and glutamine. The target protein false discovery rate (FDR) was set to <0.05. Label-free relative protein quantitation on the precursor level was performed using the same software platform, which aligns chromatograms, extracts ion peaks and integrates them to compare peptide/protein spectral abundance along the different datasets. We enabled 30% of replicate features and the ANOVA hypothesis test to determine the statistical significance associated with the protein ratio calculation. The obtained *p* value was adjusted using the Benjamini–Hochberg correction for the FDR. Normalization was performed using the total peptide amount on controls average.

### 4.7. Bioinformatic Analysis

The proteomics data were analyzed by the Search Tool for Retrieval of Interacting Genes/Proteins (STRING) database version 11.0 that can construct protein–protein interaction (PPI) networks and can compute a functional enrichment analysis [63]. The networks were built using the following parameters: Mus musculus as species, a minimum required interaction score >0.15, and the active interaction sources used were text mining, experiments, databases, and co-expression. The Gene Ontology (GO) analysis was conducted to classify the proteins identified and quantified in the experimental groups from a Biological Process (BP) standpoint. FDR < 0.05 was selected as the cutoff criteria to identify the enriched functional categories and pathways. The Cytoscape software (version 3.8.2.) [64] was used to visualize the PPI network generated by STRING. In addition, ClueGO [65] and CluePedia [66] apps were used to enhance the interpretation of biological data. InteractiVenn tool [67] was used to represent the overlap of differentially expressed proteins (DEPs) between the datasets obtained at 3 and 7 days post-MI. Principal component analysis (PCA) was performed using Proteome Discoverer 2.4. PCA was used for visualizing the differences between all experimental groups based on the abundance of proteins involved in a BP. PCA and volcano plots showing differential abundances of proteins were performed using OriginPro, version 2021b (OriginLab Corporation, Northampton, MA, USA). In this paper, we used the nomenclature of the universal protein knowledgebase (UniProtKB) for the organism Mus musculus. The protein symbols used throughout the paper are the same as the gene symbol but are not italicized, and all are upper case.

### 4.8. Real-Time PCR

Total RNA (3 µg) obtained from left ventricle was reverse transcribed to cDNA using a SuperScript™ IV First-Strand Synthesis System (Thermo Fisher Scientific) according to the manufacturer’s instructions. Real time-PCR (RT-PCR) was performed in triplicate in 10 μL reactions volume with PowerUp SYBR Green PCR Master Mix (Applied Biosystems, Waltham, MA, USA), 100 ng first-strand cDNA and 0.2 μM stock concentration of each gene-specific primer. Quantification was performed by a LightCycler 480 instrument (Roche, Basel, Switzerland), and the endogenous housekeeping Tbp gene coding for TATA box-binding protein was used as internal control. The specificity of each assay was validated by dissociation curve analysis. Relative quantitative gene expression was performed with the LightCycler^®^ 480 software using the Ε (Efficiency)-Method.

The primer sequences used were as follows: Nucleophosmin (Npm1) forward, 5′-GGAGGCAGTTGTTTTCCGTC-3′ and reverse, 5′-TTGTCAGCCTTTAGTTCACAGC-3′; Natriuretic peptides A (Nppa) forward, 5′-ATCTGATGGATTTCAAGAACCTGC-3′ and reverse, 5′-CTCGGGGAGGGAGCTAAGTG-3′; Protein S100A9 (S100a9) forward, 5′- TGGCAACCTTTATGAAGAAAGAGAA-3′ and reverse, 5′-CAGCCTTTGCCATGACTGTG-3′; Myosin-6 (Myh6) forward 5′-CAGAGATTTCTCCAACCCAGGA-3′ and reverse, 5′-GTCATTCTGTCACTCAAACTCTGG-3′; Protein S100-A8 (S100a8) forward, 5′-GTCCTCAGTTTGTGCAGAATATAAA-3′ and reverse, 5′-GCCAGAAGCTCTGCTACTCC-3′; Telethonin (Tcap) forward, 5′-CCGGAAGAGGGATGCTCCT-3′ and reverse, 5′-CTGGTACGGCAGCTGGTATT-3′; Sarcoplasmic/endoplasmic reticulum calcium ATPase 2 (Atp2a2) forward, 5′-TGGAACCTTTGCCGCTCATTTT-3′ and reverse, 5′-GAGCAGGAAGATTTGGTGGCA-3′; Leiomodin-2 (Lmod2) forward 5′-GACCGAAACCTTCCTGTGGG-3′ and reverse 5′-TCTTCTTCTGCAACCTTTCCAC-3′; Nucleolar protein 3 (Nol3) forward 5′-GGGACTATCCGAAACGCTC-3′ and reverse 5′-GCAATGTCTCTACCAGCCG-3′; BAG family molecular chaperone regulator 3 (Bag3) forward 5′-TTCGAGCCGCTTCTCCATTC-3′ and reverse 5′-CTCGATGGGTCATGGGCTGA-3′; Cellular tumor antigen p53 (P53) forward, 5′-GCTCACCCTGGCTAAAGTTCT-3′ and reverse, 5′-GTCTTCGGAGAAGCGTGACA-3′; Moesin (Msn) forward 5′-GATTGGCTTCCCGTGGAGTG-3′ and reverse 5′-CCGCTTGTTAATCCGAAGCC-3′; Y-box-binding protein 3 (Ybx3) forward 5′-CCAGTATCGCCCTCCATACC-3′ and reverse 5′-GATGAGCCTGGAGCTGTGTT-3′; Splicing factor, proline- and glutamine-rich forward and reverse (Sfpq) forward 5′-AAGCTGGCCCAGAAGAATCC-3′ and reverse 5′-TCTCATCAGATCTTGGCGCA-3′; Na(+)/H(+) exchange regulatory cofactor NHE-RF1 (Slc9a3r1) forward 5′-TGTGGAGGTCAATGGTGTCTG-3′ and reverse 5′-ATCTCTCCATTGCTGAAGGGC-3′; Endoplasmic reticulum resident protein 29 (Erp29) forward 5′-CGACACCCAGTACCCCTATG-3′ and reverse 5′-CATGTTCAGCTTGTCGCCAT-3′; Cysteine and glycine-rich protein 3 (Csrp3) forward 5′-TGGAGGTCTCTGCTCCAACTC-3′ and reverse 5′-GCACTGGATTTCTTCTGCATGG-3′.

### 4.9. Western Blot Assays

The protein extracts (20 µg/lane) were separated by electrophoresis on a 10% sodium dodecyl sulfate polyacrylamide gel (SDS-PAGE) and then transferred onto nitrocellulose membranes. The membranes were blocked with Tris-buffered saline (TBS) containing 2% BSA for 1h at room temperature and incubated with antibodies against nucleophosmin (1:500), natriuretic peptide A (1:500), and moesin (1:500) at 4 °C overnight, followed by incubation with horseradish peroxidase (HRP)-conjugated secondary antibodies (1:4000) at room temperature for 1 h. The immune complexes were detected using the ECL chemiluminescence kit and visualized in a GE Healthcare ImageQuant™ LAS 4000 system. Densitometric semi-quantification was performed using the Image J software (National Institutes of Health). To normalize target protein level, the total protein staining with Ponceau S was used after transfer.

### 4.10. Statistics

Statistical analysis was performed in GraphPad Prism (version 8, San Diego, CA, USA). The Student’s t test was utilized to compare two experimental groups. ANOVA hypothesis test was used for multiple comparison statistical analysis. Data are expressed as mean ± standard deviation (SD). *p* < 0.05 was considered to be statistically significant.

## 5. Conclusions

The novel data resulting from this study demonstrate that the short-term pharmacological blockade of the pro-inflammatory alarmin S100A9:

(i) downregulates the expression of proteins involved in leukocytes recruitment and has important beneficial consequences on post-ischemic myocardial repair and recovery;
(ii) favorably modulates the levels of multiple anti-apoptotic and pro-apoptotic proteins involved in p53-related pathways;
(iii) maintains higher expression levels of cardiac structural proteins and inhibits several mediators previously found to drive abnormal compensatory cardiac recovery;
(iv) reduces the markers of cardiac stress, such as NPM1, CSRP3, NPPA, TPT1 and NOL3.

The cardioprotective effects disclosed by this study provide detailed mechanistic explanation for the important cardiac functional gain induced by the short-term S100A9 blockade, supporting this therapy as a potential future treatment for acute MI.

## Figures and Tables

**Table 1:** Parameters of protein inference associated with the 46 differentially expressed proteins (DEPs) discussed in the manuscript. Confidence attributes, such as the number of unique peptides, and Sequest score pertaining to each protein are presented.

		3 Days Post-MI		7 Days Post-MI	
Uniprot Accession Key	Protein Description	# Unique Peptides	Sequest Score	# Unique Peptides	Sequest Score
P62082	40S ribosomal protein S7	-	-	9	346.06
Q9CXW4	60S ribosomal protein L11	-	-	7	192.95
P48962	ADP/ATP translocase 1	-	-	10	1226.26
Q9JI91	Alpha-actinin-2	-	-	11	266.49
Q62167	ATP-dependent RNA helicase DDX3X	3	80.25	-	-
Q9JLV1	BAG family molecular chaperone regulator 3	25	2680.89	30	4404.55
Q01339	Beta-2-glycoprotein 1	24	2855.16	-	-
P45591	Cofilin-2	8	1206.06	14	1043.4
P50462	Cysteine and glycine-rich protein 3	-	-	23	5298.01
P57759	Endoplasmic reticulum resident protein 29	-	-	5	105.24
P63242	Eukaryotic translation initiation factor 5A-1	10	851.31	13	1115.78
O09164	Extracellular superoxide dismutase [Cu-Zn]	-	-	11	404.16
P11352	Glutathione peroxidase 1	-	-	10	966.72
P14602	Heat shock protein beta-1	19	6349.35	20	8314.62
P61979	Heterogeneous nuclear ribonucleoprotein K	-	-	28	3133.52
P09055	Integrin beta-1	2	1287.76	-	-
Q3UHZ5	Leiomodin-2	-	-	4	64.35
P34884	Macrophage Migration inhibitory factor	4	649.78	6	1206.71
Q9CQ92	Mitochondrial fission 1 protein	-	-	4	125,56
P26041	Moesin	2	13.65	-	-
Q02566	Myosin-6	-	-	121	14,283.36
P70441-1	Na(+)/H(+) exchange regulatory cofactor NHE-RF1	-	-	4	49.87
Q8K2C6	NAD-dependent protein deacylase sirtuin-5, mitochondrial	-	-	3	73.49
Q9ERS2	NADH dehydrogenase [ubiquinone] 1 alpha subcomplex subunit 13	-	-	6	533.68
P05125	Natriuretic peptides A	-	-	7	558.71
Q99K48	Non-POU domain-containing octamer-binding protein	-	-	5	45.36
Q9D1X0	Nucleolar protein 3	7	1464.15	10	1288.64
Q61937	Nucleophosmin	-	-	15	4324.81
Q99KR7	Peptidyl-prolyl cis-trans isomerase F, mitochondrial	-	-	16	3735.42
Q9QYS9	Protein quaking	4	60.89	6	217.52
P27005	Protein S100-A8	4	2554.06	-	-
P31725	Protein S100-A9	6	1466.6	8	1068.22
Q99LX0	Protein/nucleic acid deglycase DJ-1	-	-	13	2671.72
P23506	Protein-L-isoaspartate(D-aspartate) O-methyltransferase	-	-	9	798.88
O55143-1	Sarcoplasmic/endoplasmic reticulum calcium ATPase 2	-	-	9	168.54
Q8VIJ6	Splicing factor, proline- and glutamine-rich	-	-	10	612.84
P08228	Superoxide dismutase [Cu-Zn]	-	-	21	8472.76
P09671	Superoxide dismutase [Mn], mitochondrial	11	4230.63	15	3949.1
O70548	Telethonin	9	1360.44	-	-
A2ASS6	Titin	-	-	7	47.42
P63028	Translationally-controlled tumor protein	-	-	9	2521.48
P58771-1	Tropomyosin alpha-1 chain	-	-	7	74,845.96
Q60930	Voltage-dependent anion-selective channel protein 2	11	1464.28	-	-
O88342	WD repeat-containing protein 1	-	-	4	21.46
O70373	Xin actin-binding repeat-containing protein 1	9	298.66	25	1030.8
Q9JKB3-1	Y-box-binding protein 3	5	1618.17	-	-

## Data Availability

The Mass spectrometry proteomics data were deposited into the PRIDE [68] partner repository via ProteomeXchange with the dataset identifier PXD033683.

## Supplementary Materials

The following supporting information can be downloaded at: https://www.mdpi.com/article/10.3390/ijms23095289/s1.
