# Supplementary material for: Short-Term Blockade of Pro-Inflammatory Alarmin S100A9 Favorably Modulates Left Ventricle Proteome and Related Signaling Pathways Involved in Post-Myocardial Infarction Recovery
Source: Int J Mol Sci. 2022 May 9;23(9):5289. doi: 10.3390/ijms23095289 (PMC9103348; doi:10.3390/ijms23095289)
Supplement: Supplementary file 1 [file ijms-23-05289-s001.zip › ijms-1700250-Supplementary Material.pdf]

Short-Term Blockade of Pro-Inflammatory Alarmin S100A9 Favorably Modulates Left Ventricle Proteome and Related Signaling Pathways Involved in Post-Myocardial Infarction Recovery

Raluca Maria Boteanu [1], Viorel-Iulian Suica [1], Elena Uyy [1], Luminita Ivan [1], Aurel Cerveanu-Hogas [1], Razvan Gheorghita Mares [2], Maya Simionescu [1], Alexandru Schiopu [2,3] and Felicia Antohe [1,*]

[1] Department of Proteomics, Institute of Cellular Biology and Pathology "N. Simionescu" of the Romanian Academy, 050568 Bucharest, Romania; raluca.haraba@icbp.ro (R.M.B.); viorel.suica@icbp.ro (V.-I.S.); elena.uyy@icbp.ro (E.U.); luminita.radulescu@icbp.ro (L.I.); aurel.cerveanu-hogas@icbp.ro (A.C.-H.); maya.simionescu@icbp.ro (M.S.)

[2] Department of Pathophysiology, University of Medicine, Pharmacy, Sciences and Technology of Targu Mures, 540142 Targu Mures, Romania; razvan.mares@umfst.ro (R.G.M.); alexandru.schiopu@med.lu.se (A.S.)

[3] Department of Clinical Sciences Malmö, Lund University, 21428 Malmö, Sweden

* Correspondence: felicia.antohe@icbp.ro; Tel.: +40-213-192-737

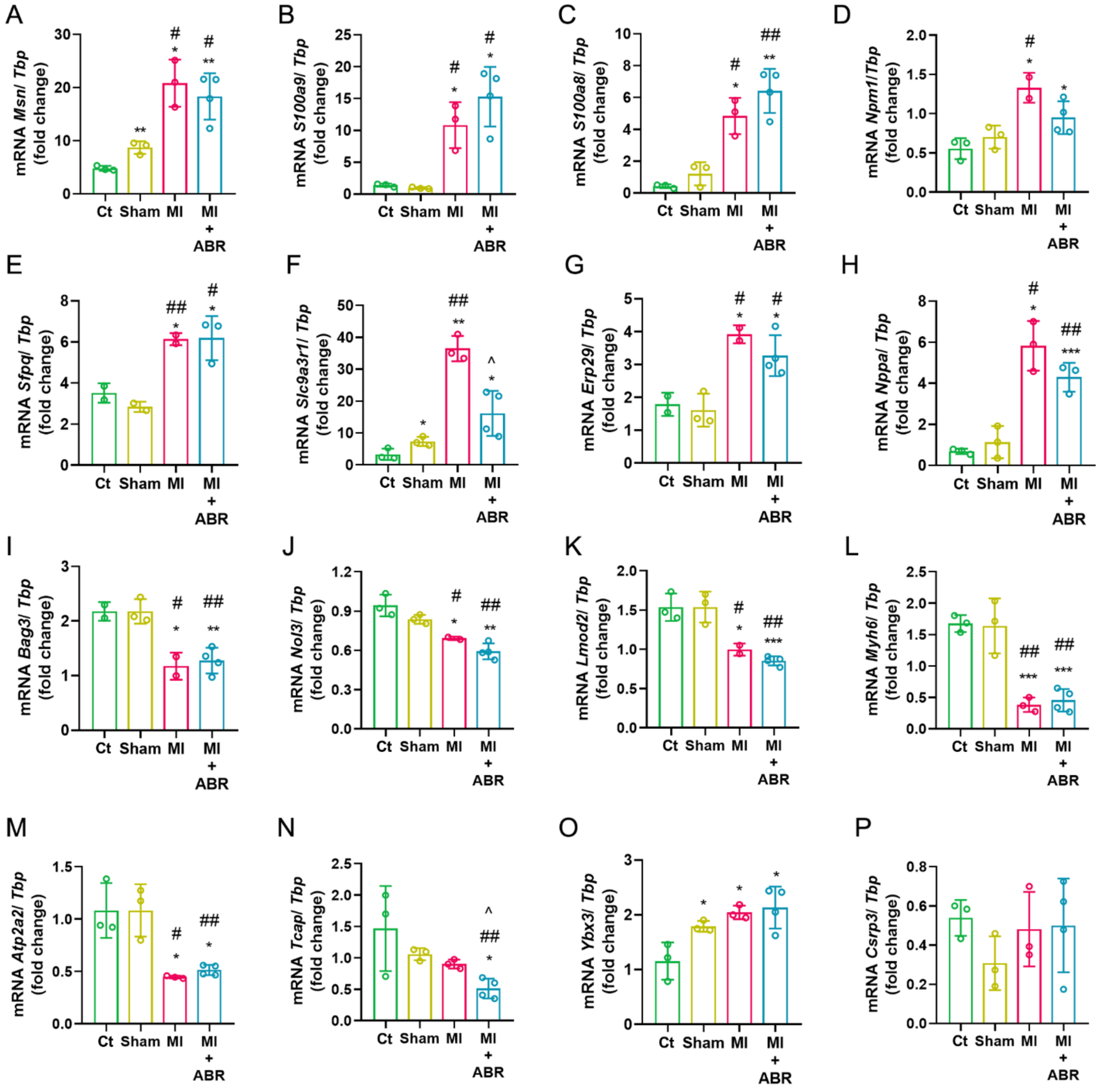

Figure S1. Gene expression profile of the identified differentially expressed proteins (DEPs) at 3 days post-MI mRNA expression levels of *Msn, S100a9, S100a8, Npm1, Sfpq, Slc9a3r1, Erp29, Nppa, Bag3, Nol3, Lmod2, Myh6, Atp2a2, Tcap, Ybx3* and *Csrp3* in the left ventricle of control Ct, Sham, MI and MI+ABR groups were quantified by real-time PCR, using *Tbp* as internal control (**A–P**). Data are presented as mean ± SD; $p^{*} < 0.05$, $p^{**} < 0.01$, $p^{***} < 0.001$ vs. Ct; $p\# < 0.05$, $p\#\# < 0.01$ vs Sham; $p^{\wedge} < 0.05$ vs. MI; *p*-values were calculated using Student's t test.

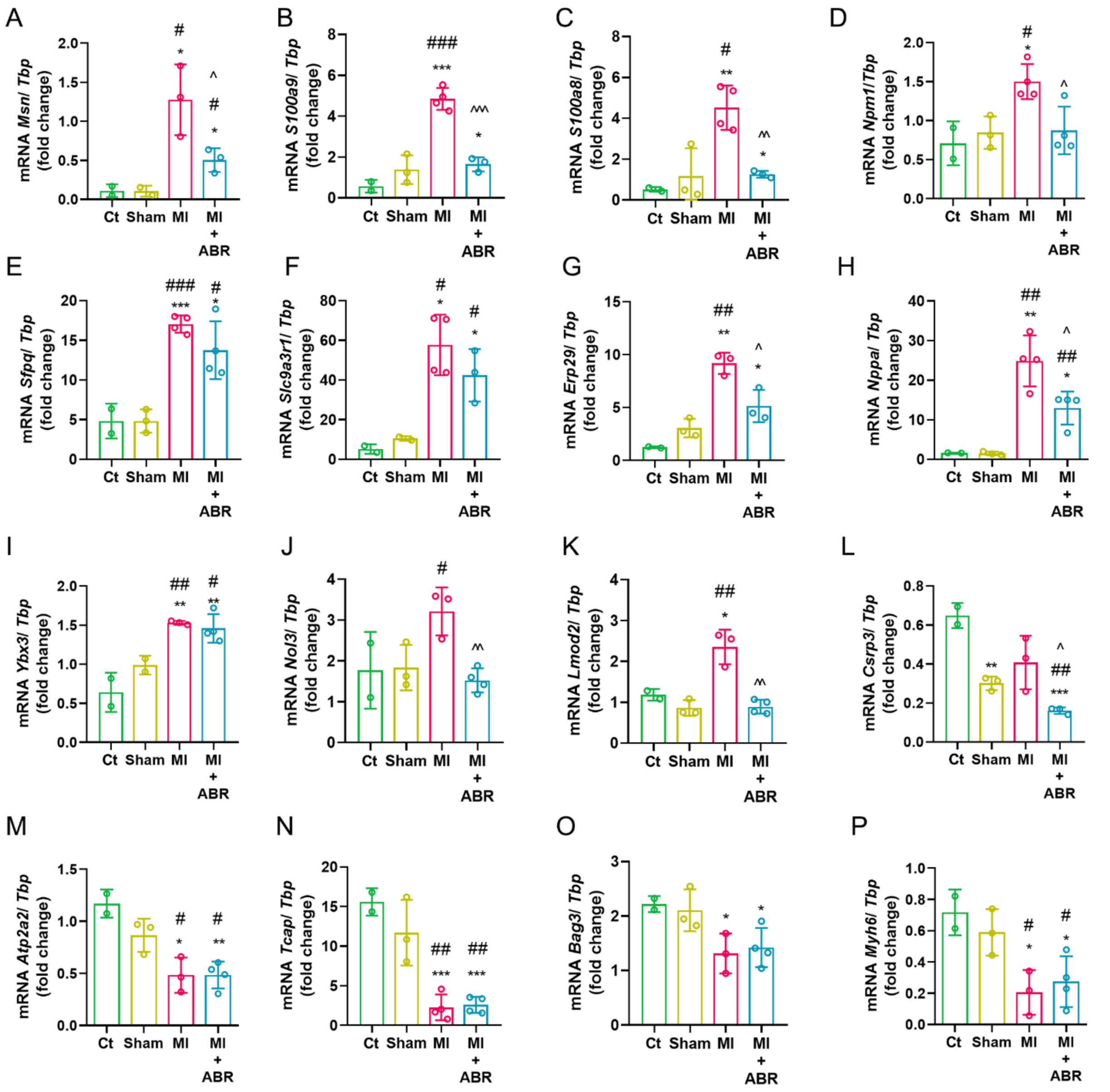

Figure S2. Gene expression profile of the identified DEPs at 7 days post-MI mRNA expression levels of *Msn, S100a9, S100a8, Npm1, Sfpq, Slc9a3r1, Erp29, Nppa, Ybx3, Nol3, Lmod2, Myh6, Atp2a2 Tcap, Bag3* and *Csrp3* in the LV of control Ct, Sham, MI and MI+ABR groups were quantified by real-time PCR, using *Tbp* as internal control (**A–P**). Data are presented as mean ± SD; $p^* < 0.05$, $p^{**} < 0.01$, $p^{***} < 0.001$ vs Ct; $p\# < 0.05$, $p\#\# < 0.01$ vs. Sham; $p^\wedge < 0.05$, $p^{\wedge\wedge} < 0.01$, $p^{\wedge\wedge\wedge} < 0.001$ vs MI; *p*-values were calculated using Student's t test.
